# Circularization and Ribosome Recycling: From Polysome Topology to Translational Control

**DOI:** 10.3390/ijms27031251

**Published:** 2026-01-27

**Authors:** Zhanna A. Afonina, Konstantin S. Vassilenko

**Affiliations:** Institute of Protein Research, Russian Academy of Sciences, 142290 Pushchino, Russia

**Keywords:** eukaryotic translation, polysome, translation reinitiation, mRNA cyclization, ribosome recycling

## Abstract

It has been known for decades that eukaryotic cellular mRNAs are frequently translated by multiple ribosomes organized into polysomes of diverse topology, including circular arrangements. The closed-loop model, in which the 5′ cap and 3′ poly(A) tail are bridged by initiation factors, provided a mechanistic basis for mRNA circularization and suggested that the spatial proximity of termini facilitates ribosome recycling. Various biochemical, structural, and imaging approaches—including electron microscopy, atomic force microscopy, cryo-electron tomography, and single-molecule fluorescence—have since demonstrated that polysomes indeed adopt compact and heterogeneous conformations, with circular assemblies representing a significant fraction. Although direct visualization of ribosome recycling remains technically challenging, ribosome turnover experiments, kinetic analyses and modeling support the concept of closed-loop-assisted reinitiation (CLAR), whereby terminating ribosomes are re-utilized to sustain translation efficiency. Together, the findings suggest that mRNA circularization is a dynamic and regulated state that enhances protein synthesis under specific conditions, while linear or modular polysome architectures may dominate in others. Understanding the balance between these modes of translation remains central to elucidating the interplay between mRNA topology, ribosome dynamics, and translational control.

## 1. Introduction

Over six decades ago, pioneering studies in reticulocyte cells and lysates revealed that individual mRNA molecules can be translated simultaneously by multiple ribosomes, forming ribosomal clusters termed polyribosomes or polysomes [1,2]. Electron microscopy showed that polyribosomes adopt diverse spatial arrangements—including double-row, spiral, and circular configurations—with short circular polysomes comprising 4 to 6 ribosomes being particularly common [1,2,3,4]. In this review, we defined mRNA chains with adjacent 5′ and 3′ ends, together with their associated polysomes, as topologically circular, whereas all others were considered topologically linear. Although the basis for circular polysome topology was initially unclear, it was hypothesized to enhance translation by “recycling” terminating ribosomes, thereby preventing their dissociation from the mRNA [5].

Subsequently, the closed-loop model of mRNA circularization—bridging the 5′ and 3′ termini—was proposed [6] (see [7] for the review), preceded by the discovery of the protein interactions underlying this topology [8,9]. The synergistic effect of the eukaryotic mRNA 5′ cap and 3′ poly(A) tail in promoting translation [10,11,12] reinforced the idea that physical proximity between mRNA termini has functional consequences for translational efficiency. Direct evidence of such interactions came from the discovery of a physical association between the G subunit of eukaryotic Initiation Factor eIF4F (eIF4G) and the Poly(A)-Binding Protein (PABP) in yeast [13,14], plants [15], and mammals [16] (see [17] for the terminology). These findings established that the capped 5′ end and polyadenylated 3′ end of mRNA are held together through a chain of RNA–protein and protein–protein interactions (5′ cap/eIF4E/eIF4G/PABP/3′ poly(A)) that involves the cap-binding E subunit of eIF4F.

Finally, direct visualization of RNA cyclization was achieved using atomic force microscopy: a model capped and polyadenylated RNA formed looped structures upon incubation with yeast eIF4F (eIF4E and eIF4G) and PABP, whereas omission of any component or disruption of eIF4E or PABP binding prevented loop formation [14]. Moreover, the formation of mRNA loops mediated by eIF4F and PABP was subsequently demonstrated in vivo [18].

It is worth noting that electron micrographs of endoplasmic reticulum membranes frequently reveal polysomes composed of widely spaced ribosomes arranged at uniform intervals [19,20,21]. While circular and hairpin-like arrangements have been described, the predominant forms were G-shaped or spiral, which do not indicate the convergence of mRNA termini. In any case, the morphology of membrane bound polysomes is dictated not by the topology or shape of the translated mRNA, but by the predetermined positioning of specific membrane receptors that interact with either the nascent signal peptide or the large ribosomal subunit, although some authors have suggested that the ends of the mRNA may be brought into proximity.

Also significant is the fact that mRNA cyclization extends beyond the canonical 5′–3′ interactions mediated by the cap/eIF4E/eIF4G/PABP/poly(A) chain. For instance, histone-encoding mRNAs, which lack a poly(A) tail, contain a conserved 3′ stem-loop (SL) that engages the cap-associated translation machinery through the SL/SLBP/MIF4G(SLIP1)/eIF3/eIF4F bridge, thereby promoting mRNA circularization and efficient translation [22,23]. Similarly, many viral mRNAs that are uncapped and/or non-polyadenylated achieve circular topology via specific structural elements located near the 5′ or 3′ ends, or through viral proteins that substitute components of the canonical circularization bridge (see [24] for review).

The establishment of the closed-loop model [6] stimulated extensive research, primarily employing in vitro translation systems, which provided compelling evidence of its impact on translation—manifested in altered polysome morphology, involvement of the mRNA 3′ tail in initiation, etc. [25,26,27,28]. The above-mentioned findings on the synergistic enhancement of protein synthesis by the 5′ cap and 3′ poly(A) tail, together with computer modeling [29,30], indicated that circularization elevates translation efficiency by increasing the overall initiation rate. This, in turn, has given rise to the belief that the spatial proximity of the 5′ and 3′ termini facilitates re-utilization of terminating ribosomes (or their small subunits) on the same mRNA molecule, i.e., should lead to what can be termed as the functional cyclization of messenger RNAs [28,31,32,33] or closed-loop-assisted reinitiation (CLAR) [34].

In light of this hypothesis, two key questions arise:

1. Do the circular agglomerates of translating ribosomes observed in EM experiments correspond to mRNAs adopting a closed-loop conformation? In other words, is the interaction of mRNA termini responsible for the formation of circular polyribosomes? The answer is less straightforward than it may appear. A translating ribosome bends the mRNA chain, and successive kinks, together with specific ribosome–ribosome interactions, can generate local mRNA looping. Thus, ring-shaped ribosome arrangements may represent their intrinsic feature and, in principle, could be formed at any part of an mRNA chain independently of the mutual position of its ends (an idea originally proposed by Christensen and Bourne [21]).

2. Does direct interaction of mRNA ends and physical circularization of translating polysomes stimulate protein synthesis by re-recruiting ribosomes that have completed translation of the same transcript? Although this model has gained wide acceptance, it has not been conclusively proven by direct experiments, largely due to the technical challenges of monitoring individual translating ribosomes. Nevertheless, several lines of evidence lend support to this view.

This review focuses on studies that have sought to address these questions using diverse biochemical, biophysical, and structural approaches.

## 2. Morphology of Polysomes and mRNA Topology

### 2.1. Electron Microscopy Approaches

#### 2.1.1. Classical EM Visualization of mRNA Ends

**Figure 1:**
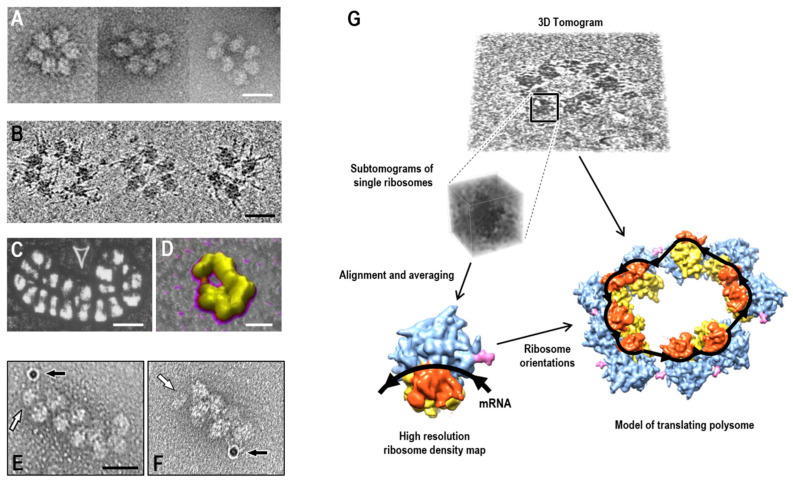
Electron microscopy approaches to polysome morphology and structure. Representative micrographs obtained by (**A**) negatively stained specimen of polysomes from wheat germ cell-free translation system (adapted from [36]); (**B**) cryo-electron tomography showing longitudinal sections of reconstructed tomograms of HeLa cell lysate (adapted from [37]); (**C**,**D**) atomic force microscopy (adapted from [38,39]). (**E**,**F**) Visualization of mRNA termini in negatively stained double-row polysomes using electron-dense markers: a 10 nm gold particle at the 3′ end (black arrow) and the initiating 40S subunit at the 5′ end (white arrow) (adapted from [40]). Scale bars, 50 nm. (**G**) Schematic representation of cryo-electron tomography mRNA path tracing in polysomes by fitting a reference ribosome model into subtomograms. Black arrows indicate evaluated trajectory of the mRNA chain. The 40S head domains are shown in orange, the body domains—in yellow, and the 60S subunit—in blue.

Early hypotheses regarding mRNA circularity emerged from analyses of classical electron microscopy images of negatively stained or metal-shadowed specimens (Figure 1A). In addition to clearly circular polyribosomes, tightly packed double-row arrangements—abundant in eukaryotic cells and cell-free translation systems—were also frequently interpreted as circular, with a single mRNA strand looping back in opposite directions to form parallel rows [26,35,36]. However, such interpretations remained provisional, since the trajectory of an mRNA chain cannot be resolved in conventional electron micrographs. The use of electron-dense markers at both ends of an mRNA, clearly visible in micrographs, could eliminate this ambiguity. If the markers appear close together within a linear structure, they would indicate a looping topology of the chain, whereas markers located at opposite ends would unambiguously demonstrate a linear topology (Figure 1E,F).

In the study by Afonina et al. (2013) [40], one end of the mRNA was labeled with an electron-dense tag to investigate its topology within double-row polysomes. Polyribosomes were assembled on an mRNA whose 3′ end had been chemically modified with a fluorescein derivative, enabling subsequent labeling with a 10 nm gold particle conjugated to fluorescein-specific antibodies. The 5′ end of the mRNA was identified in electron micrographs by the presence of a bound 40S ribosomal subunit. This approach revealed that mRNA within double-row polyribosomes can adopt either a linear topology—with the 5′ and 3′ ends positioned at opposite termini of the polysome—or a circular topology, with both ends located at the same terminus.

In another study, proteins associated with mRNAs in polyribosomes isolated from H1299 cells were labeled with electron-dense markers [41]. The authors demonstrated that the interaction between subunit h of the initiation factor eIF3 (positioned at the mRNA 5′ end) and the methyltransferase METTL3 (bound to the 3′ UTR of select cellular mRNAs) enhances translation efficiency and promotes the formation of heavier polyribosomes. To assess whether this effect was linked to mRNA circularization, they visualized the spatial proximity between 10 nm gold particles conjugated to an eIF4E-specific antibody (targeting the 5′ cap-bound initiation factor) and 6 nm particles conjugated to anti-METTL3 antibodies. The two labels were consistently detected within a distance of less than 50 nm, confirming the loop model.

#### 2.1.2. Cryo-Electron Tomography and Ribosome Orientation Mapping

Although classical electron microscopy (EM) has been used to analyze polyribosomes, the requirement to bind samples to a support followed by drying often distorts macromolecular structures. Today, cryo-electron microscopy (cryo-EM) is the predominant method for studying ribosomes and ribosomal complexes [42]. Rapid freezing of samples in amorphous ice preserves macromolecules in a near-native state. Over the past decade, cryo-electron microscopy has undergone remarkable advancements in macromolecular structure reconstruction. This progress is especially evident in single-particle analysis, which now enables the resolution of individual ribosome structures at an impressive 1.5 Å. However, this method is best suited for homogeneous and stable specimens, making it less effective for analyzing structurally diverse polyribosomes. To address the inherent variability and heterogeneity of polysomes, cryo-electron tomography (cryo-ET) was developed. By imaging the same specimen incrementally at multiple tilt angles, cryo-ET enables the reconstruction of representative three-dimensional models of a single target object. Cryo-ET analysis of polysome architecture can be carried out in situ under near-native conditions, using thin lamellae or cellular protrusions as specimens, or in cell lysates [43] (Figure 1B).

The principal limitation of cryo-electron tomography though, is its relatively low resolution, which hampers direct visualization of the mRNA molecule and precise localization of its 5′ and 3′ ends—particularly within single-stranded regions. The more discernible elements of the mRNA secondary structure are virtually indistinguishable from the ribosomal RNA expansion segments common in eukaryotes. This constraint also precludes reliable structural analysis of individual ribosomes, and to determine their precise orientations, the fitting of a reference model is to be employed. A high-resolution 3D model of the translating ribosome—typically reconstructed via single-particle analysis of the same sample or by averaging ribosome-associated cryo-ET regions—is fitted into the corresponding ribosomal densities within the tomogram using maximum cross-correlation values (Figure 1G). Given the known positions of the mRNA entry and exit sites on the translating ribosome, this approach enables tracing the path of the mRNA and estimating the likelihood that given ribosomes belong to the same polysome.

Cryo-ET analysis of the isolated polyribosomes directly within cells is often hindered by the high ribosomal density in the cytoplasm. In an in situ investigation of translating ribosomes within human glioma protrusions [44], the authors addressed this challenge not by reconstructing entire polysomes, but by analyzing the relative orientations of neighboring ribosomes. This strategy allowed them to generate statistical maps that revealed the four most prevalent configurations of ribosome pairs, two of which resembled the top-to-top contacts in bacterial polysomes [45], where ribosomes interact through the contact between 40S subunits and head domains of small subunits and the central protuberances of both ribosomes are facing a similar direction. It was shown that approximately 25–35% of the analyzed ribosomes were engaged in these major types of interactions. Three-dimensional modeling demonstrated that repeated occurrences of ribosomes in one of the preferred orientations, or combinations of different preferred orientations, yields polyribosomes with a helical or spiral architecture. These observations have been interpreted as inconsistent with the model of circular translation, suggesting that the spatial arrangement of translating eukaryotic polyribosomes may resemble that of prokaryotic polysomes [44]. However, such conclusions may be premature, as they do not fully account for the inherent limitations of in situ analysis. The construction of polysome models based on preferred mutual orientations of ribosomes remains provisional, as high ribosomal density precludes the unambiguous assignment of individual ribosomes to specific polysomes. Notably, up to 70% of ribosomes with random mutual orientations can form circular assemblies lacking a defined structure, in contrast to the ordered helical conformations. For example, in situ structural analysis of interribosomal contacts in HEK293 cell polysomes revealed two additional paired arrangements beyond the previously described top-to-top contact [44], where central protuberances in a pair of ribosomes are facing different directions [46]. The frequency of these contacts decreased following homoharringtonine treatment, indicating that they are characteristic of actively translating polyribosomes [46].

Interestingly, a recent in situ study of ribosome collision stress induced by sub-inhibitory concentrations of the elongation inhibitor anisomycin in MEF cells [47] revealed that the helical polysome class appeared only under stress conditions and was absent in untreated cells. The authors proposed that this class may arise from acute ribosome collisions that have not yet been resolved. However, a similar di-ribosome configuration was observed recently in situ in untreated HEK293A cells [48].

Recently, a new algorithm was introduced that accounts not only for the relative orientation of neighboring ribosomes but also for their absolute positions [49]. This approach enabled topological clustering of translating ribosomes and partial tracking of polysomes in tomograms of primary rat neuronal preparations. Again, 45% of classified ribosomes were assigned to clusters corresponding to a three-dimensional helical conformation. However, the authors further proposed that complete polyribosomes are composed of several independent blocks, formed by ribosomes within the same transformation clusters, which function as regulatory modules. Moreover, they theorized that the integration of certain modules, particularly di-ribosomes, may facilitate mRNA looping and thereby promote circular translation.

To address the challenge of high ribosome density, the overall architecture of pre-formed cytoplasmic polyribosomes can be examined using diluted cell lysates, where inter-polysomal contacts are reduced and do not obscure ribosome assignment. Notably, cryo-electron tomography of diluted HeLa cell lysates [37] revealed that circular polyribosomes accounted for approximately 30% of the total population, offering a counterpoint to the assumption of predominantly linear topology based on in situ analyses.

Moreover, cryo-ET study of the polyribosomal fraction isolated from MCF-7 cells by sucrose-gradient centrifugation also showed the presence of round-shaped polyribosomes [39]. Analysis of ribosome–ribosome orientations revealed, in addition to the predominant top-to-top contact, novel arrangements in MCF-7 polysomes with 40S-60S or 60S-60S major interactions, and it was supposed that the formation of circular polysomes requires a heterogeneous set of ribosomal orientations.

### 2.2. Atomic Force Microscopy of Polysomes

Atomic force microscopy (AFM) has been employed to investigate the architecture of polysomes, although the inherent limitations of the method preclude determination of the orientation of individual ribosomes [38,39] (Figure 1C,D). Specifically, the morphology of light, medium, and heavy polyribosomes from MCF-7 breast carcinoma cell purified by sucrose gradient fractionation was analyzed [39]. Three predominant structural classes defined by ribosome arrangements were described, each revealing a distinctive level of rotational symmetry. Classes with one-, two-, and three-fold symmetry were named rounded, rectangular, and triangular, respectively. Notably, rounded polysomes most closely resembled the ring-shaped polyribosomes previously observed by classical electron microscopy [1,35]. Rounded polysomes accounted for approximately 10% of the heavy fraction, and this proportion was consistent regardless of the sample preparation method, whether analyzed by AFM in liquid or after drying on a solid surface.

Under specific conditions, AFM enables the simultaneous visualization of both ribosomes and ribosome-free regions of mRNA. To achieve this, polyribosome samples are adsorbed onto mica surfaces pre-treated with Ni^2+^ ions and imaged within a liquid environment. In the study by Viero et al. [39], AFM analysis of the heavy polysome fraction revealed RNA strands of variable lengths connecting ribosomal clusters of diverse shapes, which were subjected to the classification procedure described above. The authors proposed a model in which heavy polysomes comprise ribosomal clusters—circular, triangular, or rectangular—termed “cliques”, interspersed with ribosome-free mRNA segments. Functional studies have demonstrated that the relative abundance of these polysome clusters varies with the translational status of the cell. Notably, although circular cliques resembled previously described ring-shaped polyribosomes [1,35], it was the triangular cliques that exhibited the highest translational activity.

### 2.3. Fluorescence Microscopy of mRNA Conformation

Single-molecule fluorescence imaging has rapidly become a powerful approach for probing the configuration and translational status of mRNPs in cells. Its rise has been driven by advances in super-resolution fluorescence microscopy, which enable the visualization of macromolecular structures at nanoscale resolution (reviewed in [50]). Techniques such as smFISH and SiMPull employ Structured Illumination Microscopy (SIM) [51] and Total Internal Reflection Fluorescence (TIRF) microscopy [52]. Fluorescence microscopes dedicated to single-molecule analysis often utilize multicolor imaging, permitting the simultaneous detection and spatial localization of differently labeled components of a single complex.

#### 2.3.1. smFISH Mapping of mRNA Architecture

**Figure 2:**
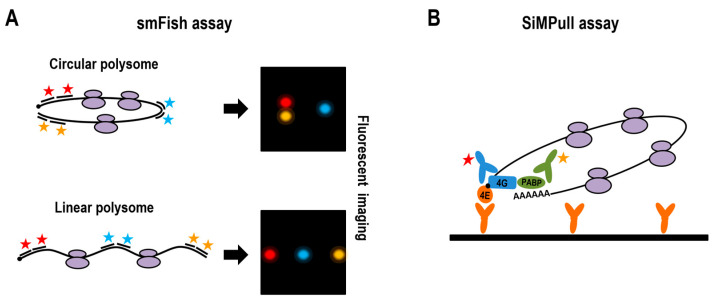
Single-molecule fluorescence imaging of polysomes. (**A**) Single-molecule fluorescence in situ hybridization (smFISH) assay, employing hybridization of short fluorescently labeled DNA oligonucleotides to defined regions of target mRNAs in fixed and permeabilized cells. Multicolor labeling enables the measurement of spatial relationships between regions of interest. (**B**) Single-molecule pull-down (SiMPull) assay, involving immuno-immobilization of translating polysomes on a support and detection of co-localized bridging factors at mRNA ends using fluorescently labeled, protein-specific antibodies. Colored stars indicate different fluorescent labels.

Single-molecule fluorescence in situ hybridization (smFISH) enables the visualization of individual mRNA molecules within cells. In this method, cells are fixed and permeabilized, followed by the hybridization of 25–50 short (∼20 nt) fluorescently labeled DNA oligonucleotides to a defined region of the target mRNA spanning ∼500–1000 nucleotides [53] (Figure 2A). Recent studies [51,54] employed smFISH to measure spatial distances between the 5′ and 3′ ends of mRNAs, both in actively translating messenger ribonucleoprotein complexes (mRNPs) and in the untranslated state under stress (HEK293 and U-2 OS cells). Specifically, the ends of AHNAK (18,836 nt), DYNC1H1 (14,361 nt) [54], MDN1 (18,413 nt), POLA1 (5486 nt), and PRPF8 (7295 nt) mRNAs [51] were labeled. These analyses revealed that longer mRNAs (>14,000 nt; AHNAK, DYNC1H1, MDN1) exhibited end-to-end distances of 130–200 nm within polyribosomes, whereas shorter transcripts (5000–7000 nt; POLA1, PRPF8) showed distances of ∼100 nm.

Upon cellular stress, however, end-to-end distances decreased to 40–50 nm as mRNAs transitioned from active to untranslated states. This compaction suggests that these transcripts are not circularized during translation; rather, polysome disassembly brings the ends closer together [51]. Notably, the mRNAs analyzed contained exceptionally long coding regions (4407–17,673 nt). Previous functional studies proposed that circular mRNP formation may depend on transcript length [31,55,56], raising the possibility that ribosome loading on long transcripts prevents convergence of their ends. A key limitation of smFISH is that it requires substantial RNA length; shorter mRNAs (<1000 nt), which are more prone to closed-loop formation, cannot be effectively analyzed.

To probe overall mRNP architecture, the central regions of AHNAK and DYNC1H1 mRNAs were also labeled [54]. These experiments demonstrated that actively translating mRNAs are far more compact than predicted by linear or hairpin models of polyribosomes. For AHNAK, the median distance between the 5′ and 3′ ends was ∼200 nm, whereas distances between either end and the central region were ∼150 nm. These values were dramatically shorter than the extended contour length (∼5.4 μm) or the estimated hairpin model size (∼2.7 μm). Similar results were obtained for DYNC1H1, indicating that extremely long mRNAs adopt highly compact conformations during translation.

Angular measurements further supported this conclusion. In non-stress conditions, most angles between vectors connecting the mRNA center to its ends were <90°, with a median of ∼60°, consistent with the assumption that these mRNAs are not linear and the ends being pulled together by intrinsic features of polyribosomes or RNA-binding proteins. Under stress conditions, angles decreased further with most of them being <45° (median ∼20°), confirming that untranslated mRNAs are even more compact.

A somewhat different analytical approach was employed in another smFISH experiments [51]. The average volume of MDN1 mRNAs in cells was also measured based on the distances between their 5′/3′ ends and the middle region, although the authors aligned the center of mass of each individual transcript and calculated the mean radius of gyration. The global size of the mRNP was found to be ∼74 nm, a value corresponding to polysomes with ribosome occupancy of 6–10. With a contour length being comparable to that of AHNAK (≥5 μm), MDN1 mRNPs also exhibited striking compactness.

#### 2.3.2. SINAPS Imaging of Translating Polysomes

Compactness of translating polyribosomes was also assessed using single-molecule imaging of nascent peptides (SINAPS [57]). This method tracks nascent proteins synthesized from mRNAs encoding multi-epitope tags. Synthesis of a multi-FLAG tag was visualized with fluorescently labeled antibody fragments, while the 3′-UTR was detected via fluorescent MS2 coat protein binding. Diffraction-limited fluorescence spots from polysomes were localized by Gaussian fitting to achieve super-resolution. Across three mRNAs with coding sequences ranging from ∼450 to ∼4500 nt, the average distance between nascent peptides and the 3′-UTR remained ∼85 nm. These findings indicate that polysomes adopt a globular rather than extended configuration.

#### 2.3.3. Estimating Circular Polysome Frequency with SiMPull Assay

The single-molecule pull-down (SiMPull) assay was employed to estimate the proportion of cellular mRNAs adopting a cyclized conformation by assessing colocalization of polysome-associated proteins that bridge the 5′ cap and 3′ poly(A) tail, namely eIF4E, eIF4G, and PABP. In this approach, target complexes were immobilized on a surface via one of their constituent proteins, and the presence of additional components was detected using fluorescently labeled, protein-specific antibodies, thereby enabling the quantification of relative protein abundance within captured complexes [58] (Figure 2B). In a recent study, polysomal fractions from HEK293FT cells pre-fixed with 1% formaldehyde were immobilized on surfaces coated with either anti-eIF4E or anti-PABP antibodies and subsequently probed with fluorescent antibodies to eIF4G and PABP, or to eIF4E and eIF4G, respectively [52]. The degree of colocalization among eIF4E, eIF4G, and PABP proved relatively low—5.6 ± 0.9% for complexes immobilized via eIF4E and 2.2 ± 0.3% via PABP. These results suggest that only a small fraction of polysomal mRNAs adopt a circular configuration. However, the low frequency of bridging complexes may reflect the dynamic nature of circular polysomes, which likely undergo frequent transitions between closed (circular) and open (linear) states. Moreover, similar colocalization values obtained in the monosome fraction (5.3 ± 0.3% when immobilized via eIF4E and 2.2 ± 0.2% via PABP) support the assumption that even mRNA translated by a single ribosome can be circularized [59] and imply that the effect of overall polysome architecture on the level of mRNA cyclization measured by the SiMPull assay may be limited.

## 3. Functional Cyclization and Ribosome Recycling

The establishment of the closed-loop model [6] fostered the idea that the spatial proximity of the 5′ cap and 3′ poly(A) tail facilitates re-utilization of terminating ribosomes—or their small subunits—on the same mRNA, thereby enabling what can be termed functional cyclization of messenger RNAs [28,31,32,33]. Although this hypothesis has gained broad acceptance, direct experimental proof remains elusive, largely because of the inherent difficulty in tracking the behavior of individual ribosomes. Nevertheless, extensive research, primarily in in vitro translation systems, has yielded substantial indirect evidence for closed-loop-assisted reinitiation (CLAR, termed in [34]). Such evidence includes alterations in polysome morphology and the involvement of the 3′ tail in initiation [25,26,27,28].

### 3.1. Ribosome Turnover in Polysomes

**Figure 3:**
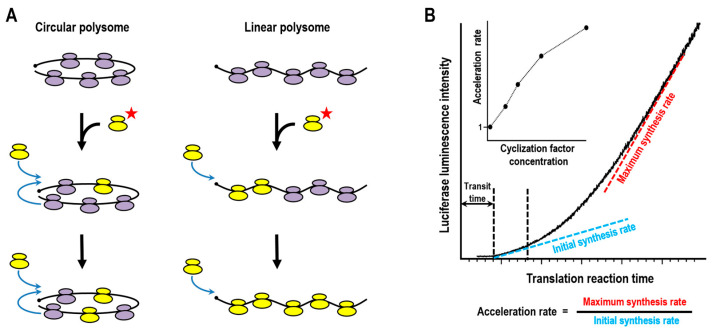
Functional assays for mRNA cyclization. (**A**) Schematic of the classical ribosome turnover assay: infusion of labeled ribosomes into an actively translating system followed by monitoring their incorporation into pre-formed polysomes. Red star indicate ribosomes with a radioactive label. (**B**) Kinetics of luciferase synthesis in a cell-free translation system, measured continuously by luminescence (adapted from [34]). Dashed lines indicate estimation of synthesis rates from the slope of the kinetic curve (see text). The ratio of maximal to initial synthesis rates was used as a measure of translation acceleration (here ~3.8). Inset: example of acceleration rate dependence on the concentration of a cyclization-promoting effector.

The most straightforward test for circular translation is to determine whether ribosomes continue to translate the same mRNA molecule in successive rounds or instead dissociate after termination to replenish the pool of free ribosomes. A classical approach involves the infusion of labeled ribosomes—either into the cytoplasm of intact cells or into an actively translating cell-free system—and subsequent monitoring of their incorporation into pre-formed polysomes (Figure 3A). By comparing the rate of ribosome exchange with the duration of a single translation cycle (the so-called transit time, i.e., the time required for a ribosome to traverse the entire coding sequence), one can infer whether ribosomes preferentially recycle on the same mRNA or equilibrate freely with the cytoplasmic pool.

As early as the 1960s, pioneering studies examined the behavior of small ribosomal subunits in the cytoplasm of HeLa cells using the pulse-chase approach. In these experiments, radioactive nucleotide [^3^H]uridine was added to the cell-growth medium, and after 15 min of incubation, RNA transcription was inhibited by actinomycin D [60]. These studies revealed that although the radioactive label was rapidly incorporated into cytoplasmic 40S subunits, its appearance in translating polyribosomes was delayed, requiring several hours of incubation to reach equilibrium. Comparable results were obtained when radioactive HeLa 40S subunits were introduced into an actively translating reticulocyte cell-free system [60,61]. The authors proposed a simple explanation for the slow rate of exchange: a “topographical” mechanism in which ribosomes that complete a round of translation remain spatially close to the initiation site, thereby reattaching to the same mRNA and continuing translation. This interpretation implicitly requires that the 3′- and 5′-ends of the mRNA are brought into proximity, consistent with the circularization of mRNA templates.

Subsequent work reinforced this view. In the wheat germ extract (WGE) translation system, ribosomes within heavy polysomes were found to exchange with added [^3^H]-labeled ribosomes only slowly, with complete replacement requiring approximately 40 min—equivalent to 4–5 rounds of translation [36]. This finding also suggested that most polysomal ribosomes reinitiate translation without being released into the general ribosome pool. A similar conclusion was reached upon the analysis of the kinetics of labeled histone mRNA incorporation into polysomes in nuclease-treated reticulocyte lysates [62].

Nevertheless, ribosome exchange experiments cannot distinguish between two possible scenarios: whether terminating ribosomes preferentially reinitiate on the same polysomal mRNA, or whether they simply remain in an activated state after termination and thereby outcompete new ribosomes during canonical de novo initiation. To address this, a large excess of competitor mRNA was introduced into actively translating cell-free systems containing pre-formed polysomes, with the aim of capturing ribosomes immediately after termination [36]. Strikingly, this did not trigger rapid ribosome switching to the competitor mRNA, and reporter protein synthesis remained stable for more than five rounds of translation. These results imply that the rate of reinitiation within polysomes substantially exceeds the rate of de novo initiation on free mRNA.

### 3.2. Kinetic Analyses of Closed-Loop-Assisted Reinitiation

The above-mentioned data on synergetic 5′ cap/3′ poly(A)-dependent enhancement of protein synthesis, as well as computer modeling [29,30], suggest that cyclisation of translating polyribosomes promotes a higher translation level by increasing the overall initiation rate [28,31,32,33]. If so, the boost of translation directed by a newly introduced mRNA would not be immediate but would emerge only after at least two rounds of translation, when the pioneer ribosome(s) reach the second termination/recycling stage. In in vitro systems, where exogenous mRNAs are ribosome-free at the outset, this should manifest as a delayed rise in protein synthesis. Such a distinctive kinetic signature would provide compelling indirect evidence for the functional cyclization.

The development of an in situ luciferase assay—continuous luminescence monitoring of a cell-free translation system—made these experiments possible [63]. Kinetic measurements were shown to reliably reflect the accumulation of newly synthesized luciferase, producing smooth curves with a high signal-to-noise ratio that enabled detailed numerical analysis. Using this approach, acceleration of protein synthesis during the initial rounds of mRNA translation was later demonstrated in a wheat germ cell-free system [64]. This acceleration was discussed in terms of a two-pathway initiation model: translation begins with slow initiation at the 5′ UTR, then, as ribosomes load and the polysome rearranges, a second mechanism, reinitiation by terminating ribosomes, becomes predominant. One proposed explanation is that rearranged polysomes bring the termination site into proximity with the initiation site, thereby increasing the effective initiation rate.

Subsequently, the kinetics of reporter mRNA translation in a mammalian Krebs-2 cell-free system was examined in detail using the same approach [34]. The authors observed that the rate of protein synthesis began to rise after ~18 min of reaction, corresponding to approximately twice the ribosomal transit time. Two kinetic parameters were defined: the initial synthesis rate, calculated as the average increase in luminescence during the first 5 min following detection of the first active product, and the maximum rate, determined as the steepest slope of the kinetic curve attained during the reaction (Figure 3B). The ratio of maximum to initial synthesis rates served as a measure of translational acceleration, reaching a value of ~4 for reporter constructs containing the β-globin mRNA 5′ UTR (Figure 3B). This acceleration strictly depended on polyadenylation of the template and was abolished by the addition of poly(A) RNA fragments or the m7GpppG cap analog. It turned out that efficient functional interaction between mRNA termini required 5′ and 3′ UTRs of moderate length and was further enhanced by longer poly(A) tails. Optimal acceleration was achieved with ~80 nt 5′ UTRs and ~300 nt 3′ UTRs—lengths closely matching those commonly found in mammalian mRNAs. Collectively, these findings suggest that mRNA circularization activates what is termed closed-loop-assisted reinitiation, an alternative initiation pathway. Perhaps the most unexpected observation was that the inhibitory effect of the dominant-negative eIF4A mutant R362Q—a potent blocker of ATP-dependent eIF4F/eIF4A-mediated scanning—diminished as the reaction progressed. This implies that at later stages, a subset of ribosomes can reach the start codon in an ATP-independent manner. It was suggested that the ribosomes employing CLAR are not involved in regular scanning.

## 4. Discussion

Over six decades of research into polysome architecture and mRNA topology have progressively reshaped our understanding of how translation is organized in eukaryotic cells. Early electron microscopy revealed strikingly diverse polysome morphologies—spiral, double-row, and circular—prompting the hypothesis that circular arrangements might reflect functional recycling of ribosomes. This idea matured into the closed-loop model, supported by biochemical evidence for cap–poly(A) synergy and protein bridges linking the 5′ and 3′ termini. Subsequent advances in structural biology, including atomic force microscopy, cryo-electron tomography, and single-molecule fluorescence imaging, have provided increasingly nuanced views of polysome organization, highlighting both the prevalence of compact, globular assemblies and the dynamic nature of mRNA end-to-end proximity.

Recent studies [65,66] have shown that free mRNA can adopt intramolecular secondary structures that bring its 5′ and 3′ ends into close proximity—typically within 5–7 nm. These data are in the range of values defined for contour lengths between RNA termini determined from predicted secondary structures of native mRNAs [67]. This observation suggests that mRNA may exist in a cyclized conformation even prior to, and during, the first round of translation. However, cellular RNA is never truly “free”; regardless of its translational status, it remains associated with a defined set of proteins as part of messenger ribonucleoprotein complexes (mRNPs). In mammalian cells, pre-translational mRNPs can package mRNA into linear, rod-like structures, wherein the ends are spatially separated due to interactions with specific RNA-binding proteins [68]. It is plausible that mRNA circularization occurs only after the first round of translation, once these proteins dissociate [69]. During translation, the ribosome induces local bending of the mRNA, and successive ribosomes may further kink the transcript, potentially bringing its ends into proximity—a concept originally proposed by Christensen and Bourne [21]. This spatial convergence may facilitate the formation of a closed-loop structure further strengthened by eIF4E, eIF4G, and PABP interactions. Such circular conformations are likely dynamic, fluctuating between “closed” and “open” states to permit entry of new 40S subunits into the initiation cycle. These transitions, driven by the reversible association of loop-forming factors, may help explain the relatively low co-localization of eIF4E, eIF4G, and PABP in polyribosomes [52]. New ribosomes continue to enter until the circular polyribosome exceeds an optimal size threshold, beyond which the mRNA ends diverge and the looped structure destabilizes. Cryo-electron tomography data suggest that the average size of circular polyribosomes in cells corresponds to hexasomes [37]. Given their higher translational efficiency, circular polyribosomes are likely to form preferentially on mRNAs that require robust expression. Indeed, the most actively translated mRNAs tend to have short coding sequences (typically <1000 nt; [31]), often encoding proteins needed in large quantities. Housekeeping gene transcripts, which are highly expressed across somatic cells, also exhibit shorter 5′UTRs, 3′UTRs, and coding regions on average [70], and should more effectively retain closed-loop promoting [31,55]. In contrast, studies examining end-to-end distances often employ mRNAs with long coding regions (4407–17,673 nt) [51,54], which show even greater end separation during active translation than under stress conditions. Collectively, these findings suggest that mRNA length itself may serve as a regulatory determinant of translation efficiency (concept proposed earlier by Thompson and Gilbert [31]).

Despite the widespread acceptance of the closed-loop paradigm, direct proof that terminating ribosomes reinitiate on the same mRNA remains elusive. Classical ribosome turnover experiments in cell-free systems suggested that ribosomes within heavy polysomes recycle locally rather than equilibrating with the cytoplasmic pool, and kinetic analyses indicated that reinitiation rates exceed de novo initiation. However, recent studies revealed evidence that the closed-loop conformation can be rare in living mammalian cells under normal conditions [51,54], and the predominant form of actively translated mRNA is likely not circularized. The apparent discrepancies underscore the limitations of current methodologies: while EM and AFM capture static morphologies, fluorescence provides dynamic but indirect measures of mRNA topology. Each technique illuminates a facet of polysome behavior, but none alone can resolve the full trajectory of individual ribosomes.

Taken together, the evidence supports a model in which mRNA circularization is neither obligatory nor rare, but rather a regulated and transient state that enhances translation efficiency under specific conditions. Cap–poly(A) and alternative bridging interactions, including histone stem-loops and viral elements, provide multiple routes to end-to-end proximity. Functional cyclization likely facilitates ribosome recycling, particularly on short transcripts, thereby boosting initiation rates and sustaining high protein output. At the same time, linear or modular polysome architectures may dominate in long transcripts or under stress, reflecting the adaptability of translational machinery to diverse cellular contexts [39,49].

Translational control mediated by interactions between the 5′ and 3′ ends of mRNA—manifested in the formation of a closed-loop structure—has long been recognized as a central principle of molecular biology and has inspired decades of research [71,72,73]. However, the fundamental mechanisms underlying this regulation remain incompletely understood. A key unresolved question is whether the stimulatory effect of the poly(A) tail–PABP complex arises primarily from the stabilization of the 43S pre-initiation complex at the 5′ terminus, thereby enhancing de novo initiation [13,74,75,76,77,78,79,80], or instead from the efficient recruitment of ribosomes that have just terminated translation at the stop codon of the same transcript. The latter possibility is supported by compelling evidence that functional mRNA cyclization can occur even in the absence of canonical bridging factors—for example, in histone mRNAs or in numerous viral transcripts and genomic RNAs (reviewed in [24,33,81]). These observations suggest that closed-loop-assisted reinitiation may represent a general strategy by which diverse classes of mRNAs optimize ribosome utilization and sustain high translational output.

The precise mechanism by which mRNA looping promotes reinitiation by recycled ribosomes remains unresolved. The simplest explanation is that bridging the mRNA ends increases the local concentration of recycled ribosomal subunits near the 5′ end, thereby favoring reinitiation on the same template. While this model is plausible, experimental data suggest that additional, specialized mechanisms may facilitate functional cyclization. Notably, closed-loop-assisted reinitiation displays low sensitivity to inhibitors of ribosomal scanning [34], implying unique mechanistic features.

It has been shown that the mean distance between stop codons and the nearest downstream poly(A) sites in human and mouse genes is approximately 300 nt [82], a spacing also found to be optimal for CLAR [34]. At first glance, such a substantial distance appears counterintuitive, since efficient cyclic reinitiation requires close proximity between recycled ribosomal subunits and the 5′ end of the mRNA. Given that poly(A)–PABP interactions provide the physical linkage between mRNA termini, the effective distance between the stop codon and the poly(A) tail is likely much shorter than the straightforward nucleotide count suggests. Importantly, the stop-to-poly(A) spacing may not directly reflect the length of the intervening sequence, as secondary structure elements inevitably pull together the ends of any sufficiently long RNA fragment. Moreover, termination factor eRF3 has been shown to bridge the termination/post-termination complex and PABP through specific protein–protein interactions [83,84], reinforcing the functional connectivity between termination and poly(A)-mediated regulation.

Unlike canonical initiation, CLAR may be supported by protein factors that remain bound to the 40S subunit after termination or recycling. Candidates include ABCE1, a recycling factor also implicated in initiation (reviewed in [85,86]); eIF3, which can be recruited to the termination complex and is known to promote cis-reinitiation (reviewed in [87]); and PABP, which participates in termination [84] while simultaneously modulating 5′ end-bound eIF4F and eIF4B, which, in turn, promote termination by reinforcing the binding of the termination factor eRF1, stimulating the GTPase activity of eRF3 and facilitating the dissociation of the factors after peptide release [88]. Recycling/reinitiation factors such as eIF2D and MCT-1/DENR [89,90] may also contribute. Thus, recycled ribosomes, unlike de novo entering ribosomes, may already carry components required for 5′ UTR binding, scanning, and start codon recognition, reducing dependence on the scanning factors such as eIF4F/eIF4A.

Conformational state may further enhance CLAR efficiency. To disengage from the mRNA, the recycled 40S subunit adopts an “open” conformation, potentially favorable for 5′ UTR binding and AUG selection. Whether this transition is assisted by PABP [74] or other nearby factors remains unknown. If the start and stop codons are spatially proximate, the open 40S could even load directly near the AUG, a mode normally prohibited during de novo initiation [91]. In this state, the subunit may diffuse non-directionally along the proximal 5′ UTR in an ATP-independent manner, locating the AUG by one-dimensional wandering. The time required would scale with the square of UTR length, explaining why short leaders favor efficient CLAR. Conversely, reduced efficiency on longer 5′ UTRs may reflect limited eIF4F/eIF4A-independent scanning or stabilization of secondary structures. The finding that m6A methylation within the 5′ UTR promotes cap-independent but 5′ end-dependent initiation that bypasses eIF4F [92] adds another layer. Such methylation can even enable initiation on circular RNAs [93]. METTL3-mediated mRNA looping [41] thus exemplifies a cellular mechanism in which eIF4F can be required for circularization but dispensable as the scanning motor, highlighting alternative pathways for ribosome recycling and reinitiation.

Also, biochemical and genetic studies in yeast and mammalian systems have shown that ribosome recycling after termination is not always immediate, and that ribosomes may linger on the mRNA in polysomes, thereby supporting repeated rounds of translation of the same coding sequence [94]. Regulation of recycling factors further modulates this process: inhibition of eIF6-dependent recycling in mammalian cells delays ribosome release at stop codons, consistent with ribosomes being retained on the same ORF and poised for reinitiation [95]. Similarly, perturbation of yeast recycling factors such as Tma20/Tma22 and Tma64 allows 40S subunits to remain bound after termination, reinforcing the possibility of local reinitiation on the same transcript [96]. Conceptual syntheses of termination–reinitiation mechanisms emphasize that such recycling events are not restricted to specialized contexts like uORFs or viral messages, but can occur in standard polysomes, providing a mechanistic basis for the circular translation of linear mRNAs [97].

Summarizing, we can say that future research progress will depend on integrating high-resolution structural approaches with single-ribosome tracking and kinetic analyses in living cells. Such efforts will clarify whether closed-loop-assisted reinitiation represents a universal principle of eukaryotic translation or a specialized mechanism tuned to transcript length, sequence features, and physiological state. In either case, the study of polysome topology continues to illuminate fundamental aspects of gene expression, bridging molecular architecture with translational control and underscoring the dynamic interplay between structure and function in the ribosome–mRNA complex.

## Data Availability

No new data were created or analyzed in this study. Data sharing is not applicable to this article.
